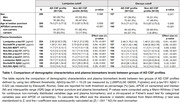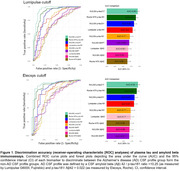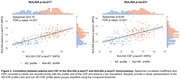# Plasma biomarkers discrimination accuracy of biologically defined Alzheimer’s disease in a memory clinic setting

**DOI:** 10.1002/alz.091345

**Published:** 2025-01-09

**Authors:** Federica Anastasi, Albert Puig‐Pijoan, Nicholas J. Ashton, Paula Ortiz‐Romero, Esther Jiménez‐Moyano, Javier Torres‐Torronteras, Armand González Escalante, Marta Milà‐Alomà, José Contador, Aida Fernández‐Lebrero, Greta Garcia‐Escobar, Nathalie Le Bastard, Alicia Nadal, Aparna Sahajan, Qinyu Hao, Bingqing Zhang, Oriol Grau‐Rivera, Henrik Zetterberg, Marta del Campo, Kaj Blennow, Marc Suárez‐Calvet

**Affiliations:** ^1^ Barcelonaβeta Brain Research Center (BBRC), Pasqual Maragall Foundation, Barcelona Spain; ^2^ Hospital del Mar Research Institute (IMIM), Barcelona Spain; ^3^ Centre for Genomic Regulation (CRG), Barcelona Institute of Science and Technology (BIST), Barcelona Spain; ^4^ Hospital del Mar Medical Research Institute (IMIM), Barcelona Spain; ^5^ Servei de Neurologia, Hospital del Mar, Barcelona Spain; ^6^ Department of Psychiatry and Neurochemistry, Institute of Neuroscience and Physiology, University of Gothenburg, Mölndal, Gothenburg Sweden; ^7^ NIHR Biomedical Research Centre for Mental Health & Biomedical Research Unit for Dementia at South London & Maudsley NHS Foundation, London United Kingdom; ^8^ Wallenberg Centre for Molecular and Translational Medicine, University of Gothenburg, Gothenburg Sweden; ^9^ King’s College London, Institute of Psychiatry, Psychology & Neuroscience, Maurice Wohl Clinical Neuroscience Institute, London United Kingdom; ^10^ Universitat Pompeu Fabra, Barcelona Spain; ^11^ Centro de Investigación Biomédica en Red de Fragilidad y Envejecimiento Saludable (CIBERFES), Madrid Spain; ^12^ Hospital del Mar Research Institute, Barcelona, Barcelona Spain; ^13^ Fujirebio Europe, Ghent Belgium; ^14^ Fujirebio Iberia, Barcelona Spain; ^15^ Alamar Biosciences, Fremont, CA USA; ^16^ Barcelonaβeta Brain Research Center, Barcelona Spain; ^17^ Department of Neurodegenerative Disease and UK Dementia Research Institute, UCL Institute of Neurology, Queen Square, London United Kingdom; ^18^ Department of Neurodegenerative Disease, UCL Institute of Neurology, London United Kingdom; ^19^ UK Dementia Research Institute, University College London, London United Kingdom; ^20^ Department of Psychiatry and Neurochemistry, Institute of Neuroscience and Physiology, University of Gothenburg, Mölndal Sweden; ^21^ Department of Psychiatry and Neurochemistry, Institute of Neuroscience and Physiology, The Sahlgrenska Academy at the University of Gothenburg, Mölndal Sweden; ^22^ Departamento de Ciencias Farmacéuticas y de la Salud, Facultad de Farmacia, Universidad San Pablo‐CEU, CEU Universities, Urbanización Montepríncipe Spain; ^23^ Department of Psychiatry and Neurochemistry, Institute of Neuroscience and Physiology, The Sahlgrenska Academy, University of Gothenburg, Mölndal, Gothenburg Sweden; ^24^ Clinical Neurochemistry Laboratory, Sahlgrenska University Hospital, Mölndal Sweden; ^25^ Barcelonaβeta Brain Research Center (BBRC), Barcelona Spain

## Abstract

**Background:**

Blood‐based biomarkers offer a non‐invasive and cost‐effective means for Alzheimer's disease (AD) detection. In this study, we performed a direct comparison of these novel biomarkers in a memory clinic population to facilitate their implementation into clinical practice.

**Method:**

We included a total of 208 patients with cognitive complaints from the BIODEGMAR cohort at Hospital del Mar (Barcelona, Spain). CSF was used as standard‐of‐truth and patients were categorized as having an AD CSF profile with two approaches: Lumipulse CSF Aβ42/p‐tau181<10.25 (N=208) or Elecsys® p‐tau181/Aβ42>0.022 (N=157). The following biomarkers were measured in paired plasma and CSF samples: Aβ42 and p‐tau181 (Lumipulse CSF<CE>, plasma<RUO>, Fujirebio), Aβ42 and p‐tau181 (NeuroToolKit (NTK) a panel of robust prototype assays (Roche Diagnostics International Ltd), and p‐tau181, p‐tau217, p‐tau231 and MAP‐T (NULISA, Alamar). P‐value and effect size of the group comparison (AD vs non‐AD CSF profiles) were calculated using a Mann‐Whitney U test. ROC curve analysis evaluated plasma biomarkers accuracy to discriminate AD from non‐AD CSF profiles. Spearman test was used to assess the correlation between plasma and CSF biomarkers.

**Result:**

Demographic information of the study cohort is reported in Table 1 for both classification thresholds. All plasma biomarkers were significantly different between the AD and non‐AD CSF profile groups, but the effect size varied among them. Specifically, NULISA p‐tau217, Lumipulse p‐tau181 and NULISA p‐tau231 reported the greatest effect sizes for the Lumipulse threshold and NULISA p‐tau217, NTK p‐tau181, Lumipulse p‐tau181 and NULISA p‐tau231 reported the greatest effect sizes for the Elecsys® threshold (Table 1). ROC curve analysis performed consistently over the two thresholds with the following discrimination accuracy (AUC) values (from highest to lowest): NULISA p‐tau217, NTK p‐tau181, NULISA p‐tau231, Lumipulse p‐tau181, NULISA p‐tau181 (Figure 1). In comparison to the p‐tau assays, Aβ42 reported modest effect size and AUCs. NULISA p‐tau217 and NULISA p‐tau231 had the highest correlation between plasma and CSF (Spearman ρ=0.75 and ρ=0.60, respectively, with both FDR corrected p‐values<0.001), Figure 2.

**Conclusion:**

In a memory clinic population, various plasma Aβ and tau plasma biomarkers, targeting different epitope and using different platforms, demonstrated high performance in distinguishing patients with biologically defined AD from those without.